# Unlocking 3D printing technology for microalgal production and application

**DOI:** 10.1007/s44307-024-00044-6

**Published:** 2024-10-08

**Authors:** Han Sun, Qian Gong, Yuwei Fan, Yuxin Wang, Jia Wang, Changliang Zhu, Haijin Mou, Shufang Yang, Jin Liu

**Affiliations:** 1 Key Laboratory of Poyang Lake Environment and Resource Utilization, Ministry of Education, and Center for Algae Innovation & Engineering Research, School of Resources and Environment, Nanchang University, Nanchang, 330031 China; 2 College of Food Science and Engineering, Ocean University of China, Qingdao, 266003 China; 3 Institute for Advanced Study, Shenzhen University, Shenzhen, 518060 China; 4 Department of Energy and Resources Engineering, College of Engineering, Peking University, Beijing, 100871 China

**Keywords:** 3D printing technology, Microalgae; Bio-ink, Environmental stress, High-value product

## Abstract

Microalgae offer a promising alternative for sustainable nutritional supplements and functional food ingredients and hold potential to meet the growing demand for nutritious and eco-friendly food alternatives. With the escalating impacts of global climate change and increasing human activities, microalgal production must be enhanced by reducing freshwater and land use and minimizing carbon emissions. The advent of 3D printing offers novel opportunities for optimizing microalgae production, though it faces challenges such as high production costs and scalability concerns. This work aims to provide a comprehensive overview of recent advancements in 3D-printed bioreactors for microalgal production, focusing on 3D printing techniques, bio-ink types, and their applications across environmental, food, and medical fields. This review highlights the benefits of 3D-printed bioreactors, including improved mass transfer, optimized light exposure, enhanced biomass yield, and augmented photosynthesis. Current challenges and future directions of 3D printing in microalgal production are also discussed to offer new insights into boosting microalgal cultivation efficiency for expanded applications.

## Introduction

Originating in the 1980s, 3D printing represents a revolutionary approach to manufacturing that utilizes computer control to precisely layer materials in three dimensions for the rapid production of intricate three-dimensional (3D) structures. Commonly referred to as “rapid prototyping” or “additive manufacturing” (Shahrubudin et al. 2019), this technology distinguishes itself from traditional manufacturing methods through its superior design flexibility and expedited production. The most notable advantage of 3D printing lies in its capacity to fabricate complex products using a single machine, obviating the need for additional components (Ligon et al. 2017). Nowadays, 3D printing is increasingly employed across various sectors, including agriculture, healthcare, and industry, for the large-scale customization and fabrication of sophisticated equipment.

As single-celled autotrophic organisms, microalgae exhibit several advantages, such as rapid growth and strong adaptability to extreme environments. They have a high photosynthetic efficiency of 2%–6%, contributing to CO_2_ sequestration at an estimate of 0.2–0.9 Gt CO_2_ yr^−1^ by 2050 (Hepburn et al. 2019). They are rich in various bioactive substances, including polyunsaturated fatty acids (PUFAs), essential amino acids, and carotenoids (Yang et al. 2023). Many microalgae species, including *Spirulina*, *Chlorella vulgaris*, *Euglena*, and *Dunaliella salina*, have been considered as rich and sustainable natural sources of protein (constituting as high as 60%–75% of their dry weight) (Caporgno and Mathys 2018). In particular, *Spirulina* is noted for its exceptional nutritional value and high protein content and shows functions in alleviating hyperlipidemia, hypertension, and kidney function deterioration (Yang et al. 2023, Yu et al. 2024). Microalgae are typically incorporated into candies, biscuits, noodles, and other foods in the form of tablets, capsules, or liquids to enhance the nutritional value (Yang et al. 2024). In addition to human nutrition, microalgae are utilized as dietary supplements in aquaculture and animal feeds, substantially improving animal weight, increasing milk production in cows, and enhancing PUFA content in milk and eggs (Amorim et al. 2021; Nagarajan et al. 2021). Furthermore, *C. vulgaris* and *Spirulina* are extensively used in skincare products, including antiaging creams, toners, sunscreens, and hair care formulations (Sun et al. 2023).

**Fig. 1:**
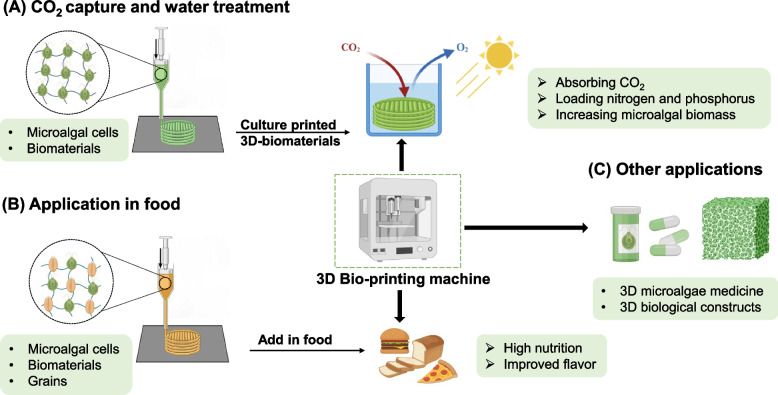
Scope of 3D bioprinting of microalgae in various fields

In the face of global challenges such as population growth, climate change, and environmental shifts, countries worldwide are encountering significant hurdles that cause food supply shortages and heightened demand for protein. Microalgae have emerged as a focal point for sustainable food and energy solutions. Compared with traditional crops, microalgae offer superior efficiency in freshwater and land use (Wang et al. 2024a). For example, producing 1 kg of protein from peas requires 18.4 m^2^ of land and 3.0 m^3^ of water on average; meanwhile, autotrophic *Chlorella* requires as little as 2.7 m^2^ of land and 0.6 m^3^ of water (Yang et al. 2024). Despite these advantages, the production of protein from microalgae entails significantly high energy inputs and comparable environmental impacts to animal protein. Producing 1 kg of protein from peas results in a global warming potential of 4–10 kg CO_2_-equivalent; *Chlorella* can emit 126.3–264.3 kg CO_2_-equivalent, comparable with beef that emits 200–750 kg CO_2_-equivalent (Yang et al. 2024). The energy consumption associated with traditional reactors further exacerbates this issue. As a solution to these challenges, alternative culture media and biological materials for reactor preparation must be explored to reduce reliance on conventional photobioreactors (PBRs) and fermenters. However, scalability and applicability remain critical hurdles. The application of 3D printing in microalgal production offers a promising avenue for the development and utilization of microalgae resources (Fig. 1). This review explores the application of 3D printing in microalgal cultivation and presents advancements in the use of printed microalgae for environmental remediation, food and medical applications, and novel material fabrication. The findings provide new insights into the further development of 3D printing in designing novel bioreactors for sustainable microalgal production.

## Traditional cultivation system for microalgal production

The three primary types of microalgal bioreactors are open ponds, photobioreactors (PBRs), and fermenters. Each system presents distinct advantages and challenges, influencing its suitability for different applications and production scales.

### Open ponds

Open ponds are outdoor, shallow ponds where microalgae are cultivated under natural sunlight. They are characterized by their simplicity, low capital costs, and scalability, making them suitable for large-scale production. However, microalgal cultivation in open ponds is highly dependent on location-specific factors, with light and temperature being critical constraints. These systems are also vulnerable to environmental fluctuations and contamination, which can impact biomass productivity and quality. In general, the biomass concentration is below 1 g L⁻^1^, and productivity is in the range of 50–70 MT ha^−1^ yr^−1^ (Branyikova and Lucakova 2021; Carlsson et al. 2007). Raceway ponds require minimal equipment, and the primary costs are associated with cooling and pollution control.

### PBRs

PBRs are closed systems that enable precise control over environmental parameters such as light intensity, temperature, and nutrient availability. Compared with open ponds, PBRs offer higher biomass productivity and greater control over culture conditions. Biomass concentrations can reach up to 10 g L⁻^1^, with productivity around 150 MT ha^−1^ yr^−1^ (Carlsson et al. 2007). Despite these advantages, PBRs are associated with high capital costs and limited scalability, which raises production expenses. For instance, the photoautotrophic cultivation of *Haematococcus pluvialis* costs $14–18 kg^−1^, and the heterotrophic production of *Chlorella sorokiniana* can be as low as $1 kg^−1^ (Yang et al. 2024). Therefore, PBRs are commonly used for producing high-value compounds such as astaxanthin and PUFAs.

### Fermenters

Fermenters, also known as closed or heterotrophic bioreactors, involve the cultivation of microalgae under controlled conditions in the absence of light using organic carbon sources. They offer several advantages, including high biomass yields, year-round production, and reduced contamination risks. Heterotrophic fermentation often employs fed-batch strategies to produce microalgal biomass at high concentrations. However, the high intracellular levels of reactive oxygen species (ROS) can impede protein synthesis, and the lack of light is detrimental to pigment accumulation (Wang et al. 2024b). In addition, the need for energy-intensive mixing and aeration increases operational costs.

### Limitations and challenges

Despite their potential, microalgal bioreactors face several limitations and challenges that impede their widespread adoption and commercialization. The capital and operational costs associated with microalgal bioreactors can be prohibitive, particularly for large-scale production. Cost-effective solutions are warranted to improve the economic viability of microalgal production. Contamination from competing microorganisms, such as bacteria and fungi, also poses a significant challenge in open ponds and PBRs. Effective contamination control strategies are essential to maintain the purity and productivity of microalgal cultivation. Microalgal growth is often constrained by nutrient availability, including nitrogen and phosphorus. Therefore, sustainable nutrient sources and recycling methods are necessary to ensure long-term viability and environmental sustainability. Scaling up from laboratory or pilot scales to commercial production introduces technical and logistical complexities. Optimizing the reactor design, operation, and process control is vital for achieving consistent and cost-effective large-scale production. Innovative technologies such as 3D bioprinting offer promising solutions to address these challenges. This technology allows for the precise fabrication of complex structures with the controlled spatial distribution of materials, enabling the development of customized reactors specifically designed to meet the needs of microalgal production.

## Innovations in 3D printing

3D printing is an advanced technique that involves the precise layer-by-layer deposition of materials to construct objects based on computer models (Kulkarni et al. 2024; Tian et al. 2017). Different from traditional manufacturing methods that are often labor-intensive and require multiple materials to build complex devices, 3D printing reduces material waste through additive manufacturing. This method supports sustainable and customized production. Beyond its use in military and aerospace applications, 3D printing has found significant utility in tissue engineering. In particular, 3D bioprinting employs biocompatible materials, cells, and support components to fabricate complex structures. It utilizes hydrogels or porous substrates as flexible, highly biocompatible “inks” to create tissue frameworks for biological applications (Tripathi et al. 2023; Zhu et al. 2016). Bioprinting is a technique that precisely deposits biomaterials containing cells or active molecules to construct intricate 3D functional tissues or artificial organs.

### Printing methods

In the evolving landscape of printing technologies, extrusion printing, thermal printing, laser-assisted printing, and acoustic-assisted printing stand out as leading methods among a diverse array of techniques (Zhu et al. 2016).

#### Inkjet printing

**Fig. 2:**
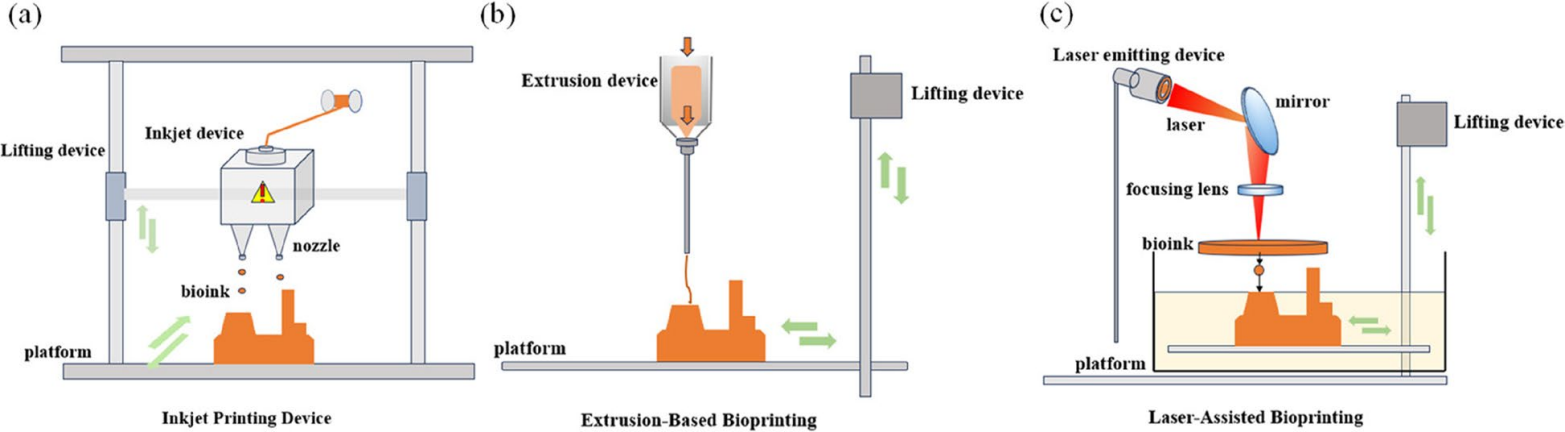
Schematic showing the key additive manufacturing techniques of inkjet printing (**a**), extrusion-based bioprinting (**b**) and laser-assisted bioprinting (**c**)

Inkjet printing operates by sequentially depositing ink droplets in layers to build up structures (Fig. 2a). This noncontact method has two modes: on-demand and continuous printing (Gudapati et al. 2016). Inkjet printing allows for the precise deposition of extremely small droplets of cells or protein solutions onto fixed positions to form specified shapes (Sadeghianmaryan et al. 2022). Its advantages include high print resolution, high throughput, high deposition accuracy, and relatively low cost. Multiple nozzles can be equipped for the simultaneous targeted printing of different cells, biomaterials, or growth factors. However, inkjet printing may suffer from issues such as directional imprecision and uneven droplet sizes. High-viscosity or concentrated bio-inks may cause nozzle clogging, and low-viscosity inks can weaken the printed material and limit the technology’s application.

#### Extrusion-based bioprinting

Extrusion-based bioprinting is one of the most widely applied printing methods. It can print high-viscosity biomaterials using air pressure or mechanical drive to extrude biomaterials as fibers onto the printing platform (Fig. 2b). This method allows for the precise placement of live cells and the combination of hydrogels to create specific-shaped devices. Its main advantages include the capability to print a wide range of biomaterials and achieve high cell density deposition (Murphy and Atala 2014). However, it has a low printing accuracy and may reduce cell viability due to the shear stress generated during extrusion (Dwivedi and Mehrotra 2020).

#### Laser-assisted bioprinting

Laser-assisted bioprinting uses lasers to deposit bio-ink onto a substrate. The equipment mainly consists of a pulsed laser source, an absorption layer, and a receiving substrate (Fig. 2c). Compared with the first two methods, its main advantage lies in its nozzle-less printing mode, which avoids issues such as nozzle clogging and mechanical damage to cells. This technique has a broad range of biomaterial choices and can print high-viscosity biomaterials. Nevertheless, it is relatively costly and may not ensure coating uniformity. In addition, the preparation process can be complicated, making it challenging to use for printing complex structural devices (Raees et al. 2023).

### Bio-ink

Bio-ink refers to biologically derived materials containing cells or active molecules. As a carrier for cells and active substances, bio-ink can significantly affect the accuracy of the printed device and is one of the key limiting factors in 3D printing. An ideal bio-ink should possess biocompatibility, printability, mechanical properties, biodegradability, ink rheological properties, and gelation characteristics (Raees et al. 2023). To date, various biomaterials with these characteristics have been widely used as bio-ink. Bio-inks for 3D bioprinting are mainly categorized into natural material-based and synthetic material-based (Table 1).

**Table 1:** Classification and applications of bio-ink

Biomaterial	Printing Method	Printing effect of bio-ink	References
**Natural Material-Based Bio-Ink**			
Chitosan, recombinant collagen	Extrusion-based printing	Bio-compatible	(Yang et al. 2022)
Alginate, honey	Extrusion-based printing	Suitable for in situ skin tissue engineering	(Datta et al. 2018)
Alginate, nanocellulose	Inkjet printing	Used for printing osteoblasts and bone tissue formation	(Im et al. 2022)
Gelatin, hyaluronic Acid	Stereolithography bioprinting	Improves cartilage tissue modeling for regenerative therapy or in vitro models	(Shopperly et al. 2022)
Gelatin, alginate, montmorillonite	Extrusion-based printing	Printed vascular scaffolds for in situ vascular tissue regeneration	(Wu et al. 2022)
Alginate	Extrusion-based printing	Stem cells exhibit a high cell viability of 88 ± 18%	(Kostenko et al. 2023)
Gelatin, suture fibers	Inkjet printing	Improves poor physical properties of natural polymer-based bio-ink	(Choi et al. 2021)
Carboxymethyl chitosan	Extrusion-based printing	Good mechanical properties, promotes cell attachment and expression of chondrogenic genes in chondrocytes	(He et al. 2020)
Gelatin, Antheraea pernyi silk fibroin nanofibers (ASFNFs)	Extrusion-based printing	Enhances shape fidelity, cell compatibility, and porosity of printed scaffolds	(Zou et al. 2022)
Methacrylated ethylene glycol chitosan	Photocuring	Survival rate above 92%, proliferation rate above 96%, hemolysis level below 2%, excellent osteogenic capability	(Chang et al. 2022)
**Synthetic Material-Based Bio-Ink**			
PEG, Low molecular weight gelatin (LMWG)	Extrusion-based printing	Improves shape fidelity, printing accuracy, maintains high cell viability, and sustained proliferation	(Piluso et al. 2021)
PEG, silk protein	Inkjet printing	Mice subcutaneously implanted for six weeks remained viable and proliferated	(Zheng et al. 2018)
PEG, nanosilica	Extrusion-based printing	Possesses mechanical properties, exhibits high vitality after cell encapsulation	(Peak et al. 2018)
PCL, magnesium hydroxide nanoparticles	Extrusion-based printing	Strong mechanical stability and bone-related bioactivity	(Alcala-Orozco et al. 2022)
Nanoparticulate PCL, alginate	Inkjet printing	Printing cell-loaded scaffolds with higher and more uniform cell seeding rates	(Kim et al. 2016)
PLA, gelatin, carboxymethyl cellulose (CMC), and alginate composite trimer	Extrusion-based printing	Strong mechanical properties, cell proliferation within the printed scaffolds, and collagen secretion increase with incubation time	(Sathish et al. 2022)
PLA, mesoporous bioactive glass	Pneumatic Extrusion	Simulates natural bone composition, increases hardness, and elastic modulus	(Pant et al. 2022)
PLA nanomatrix composite materials	Inkjet printing	Enhances osteogenic differentiation of human mesenchymal stem cells (hMSCs) and serves as a functional ligament substitute graft	(Uehlin 2012)

#### Natural material-based bio-ink

Natural materials are naturally formed within organisms and can be hydrolyzed into small molecules within organisms. They include animal-derived bioproducts, such as gelatin, collagen, hyaluronic acid, fibronectin, and silk fibroin; and nonanimal-derived materials, such as cellulose and alginates (Shamma et al. 2022). The advantages of natural materials are their good biocompatibility and biodegradability, providing an excellent 3D environment for cell growth (Wang et al. 2023a). However, these materials often have issues such as low mechanical properties and poor stability. Natural materials can be added with functional groups or combined with other biomaterials to meet the specific requirements for bio-ink. For example, the addition of nanocellulose to hyaluronic acid hydrogel-based bio-ink enables the independent printing of multilayered structures and covalent cross-linking after printing, thereby enhancing the stability and suitability for producing complex structural matrices for cell growth (Trager et al. 2023). Gelatin and alginate-based hydrogel bio-ink exhibit improved rheological properties, high printing accuracy, and good stability, thus facilitating fast wound healing (Hao et al. 2023). However, synthetic material-based bio-ink has emerged to meet the increasing demands of 3D printing for bio-ink.

#### Synthetic material-based bio-ink

Many synthetic biomaterials have been developed into bio-ink for widespread use in 3D printing. Polyethylene glycol (PEG), polyvinyl pyrrolidone (PVP), poly-caprolactone, poly-lactic-co-glycolic acid, and polyglycolic acid are commonly used synthetic materials for 3D bioprinting (Khoeini et al. 2021). Compared with natural materials, the main advantage of synthetic materials lies in their superior mechanical properties that allow for the construction of complex geometries. Furthermore, they possess biocompatibility and biodegradability similar to ceramics and metals and offer a broad range of biomaterial options. For example, PEG is an economical, highly biocompatible, water-soluble, and organic-solvent-soluble polymer used extensively in drug delivery, wound healing, and scaffold construction in tissue engineering. 3D-printed PEG scaffolds allow for the orthogonal adjustment of elastic modulus and microstructure (Soman et al. 2012). As a nontoxic, amorphous, nonionic, and highly soluble material, PVP finds wide use in tissue engineering and cosmetics. PVP-based bio-ink can reduce cell adhesion and sedimentation during the printing process. A 2.5% w/v PVP bio-ink can enhance cell viability and uniformity (Pahlevanzadeh et al. 2020). Different from natural materials, synthetic materials used as bio-ink for 3D bioprinting cannot support normal cell growth or simulate the operation of normal biological tissues.

### Customization and scalability

3D bioprinting allows for the precise customization of bioreactor structures, including the arrangement of channels, chambers, and support structures, to optimize the growth conditions for different microalgae species (Wangpraseurt et al. 2022). This customization enables researchers to design bioreactors that cater to the specific needs of microalgae cultivation, such as optimal light exposure, nutrient distribution, and gas exchange. A 3D-printed bionic coral designed to foster the growth of microalgae has been successfully developed. It enables the engineering of diverse microhabitats within the coral by employing different bio-inks to encapsulate coral photosynthetic microalgae. In addition, a customizable 3D interface is utilized to produce scalable biomimetic artificial structures by utilizing the data gathered from coral ecosystems (Wangpraseurt et al. 2020). Once the optimal bioreactor design is established, it can be easily replicated and scaled up to meet the demands of large-scale microalgae cultivation. This scalability allows researchers and industries to seamlessly transition from laboratory-scale experiments to commercial production, facilitating the widespread adoption of microalgae-based technologies. In addition, 3D bioprinting offers flexibility in design and production, allowing for rapid prototyping and iterative improvements (Parra-Cabrera et al. 2018). This flexibility also accelerates the development process and promotes innovation in microalgae cultivation techniques. When bioreactors are designed using 3D printing, the material’s ability to maintain seal under pressure is crucial. Stereolithographic techniques allow components to withstand high water pressure (Pozzobon et al. 2023). To date, studies on the utilization of 3D printing for microalgal bioreactors remain limited.

## 3D printing of microalgal bioreactors

Autotrophic microalgae possess high photosynthetic and growth efficiency and have garnered significant attention across various industries due to their substantial economic and growth potential (Ng et al. 2017). Microalgae-derived oil can be used as a sustainable replacement for diesel, and microalgal proteins as sustainable alternatives to plant proteins find wide applications in aquaculture feed and the food industry (Yarnold et al. 2019). However, production and cultivation costs must be reduced to effectively utilize the active substances in microalgae on a large scale. The preparation of photobioreactors is a key strategy for achieving this goal. Combining 3D printing with microalgae allows for design flexibility and significantly enhances the cultivation of microalgae and their photosynthetic efficiency (Table 2).

**Table 2:** 3D printing of microalgae to increase biomass and enhance photosynthetic efficiency

	Printing form	Post-printing advantages	References
Microalgae	3D printing of biomimetic coral	Microalgae density of 10^9 cells/mL	(Wangpraseurt et al. 2020)
*Chlorella vulgaris*	Cultivation with different 3D printing materials	PLA does not induce changes in reactive oxygen species	(Pozzobon et al. 2023)
*Tetraselmis suecica*	Development of 3D hydraulic vortex	Harvesting efficiency increased by 11.1 times in 13 min	(Syed et al. 2017)
*Chlamydomonas reinhardtii*	Embedding in 3D printed hydrogels	Increased growth rate and CO_2_ capture rate	(Oh et al. 2023)
*Chlorella vulgaris*	3D printing of fractal tree-like reactors	Fv/Fm approaching 0.767,	(Zhao et al. 2019a)
*Chlorella sorokiniana*	3D printing of high-density detection device	Microalgae biomass of 4.497 g L^−1^ d^−1^, CO_2_ fixation efficiency of 70.75%	(Lee et al. 2022)
*Neochloris oleoabundans*	3D printing buoyancy structure combined with flue gas CO_2_ for microalgae cultivation	CO_2_ reduction, increased cell density, and enhanced diesel production capacity	(Sung et al. 2022)

### Improved mass transfer

Various complex structures of microalgal bioreactors, such as microporous structures and nanoscale pores, can be designed and printed through 3D bioprinting, thereby increasing the mass transfer interface and improving mass transfer efficiency. In addition, various types of materials can be selected to print microalgal bioreactors, such as those with high biocompatibility and special surface properties, to enhance mass transfer efficiency and microalgal growth rate. A recent study reported the dependency of the growth, spatial distribution, and photosynthetic productivity of *Chlamydomonas reinhardtii* within 3D-shaped hydrogels on the geometry and size (Oh et al. 2023). Engineering living materials with increased CO_2_ capture rates and surface-to-volume ratios were successfully designed and printed. However, the cells in deeper layers still faced limited CO_2_ availability. The study designed 2 mm-thick hollow hemispheres to enhance gas exchange in the engineering living materials. Compared with the colonies at the edges, those located at an intermediate depth of 0.5–1.0 mm from the surface showed less increase in volume, indicating that the improved gas exchange facilitated cell proliferation. With the addition of an extra acetate in the TAP medium, the cell growth at the intermediate depth in the engineering living material hemispheres also increased. Therefore, 3D-printed bioreactors are currently not suitable for the large-scale industrial cultivation of microalgae due to the major reason of limited carbon availability (Oh et al. 2023).

### Tailored light exposure

Tailored light exposure allows precise control over the intensity, duration, and wavelength of light exposure to optimize the growth conditions for specific microalgal species. A study simulated the intricate structures of real corals to guide light into deep shaded areas, thereby enhancing the light utilization efficiency of microalgae cells (Wangpraseurt et al. 2020). In addition, light exposure can be customized spatially within the bioreactor structure to ensure uniform illumination across the microalgal culture, promoting consistent growth and productivity. A recent study designed a biogenic microalgae-laden hydrogel system for cultivating *Chromochloris zofingiensis*, which induces uniform light dispersion throughout the entire structure with a light absorption rate reaching up to 85.4% (Liu et al. 2024). Zeng et al. (2023) utilized 3D printing to create 3D porous biofilm photobioreactors (bPBRs) with nanosized organic silica particles serving as light-scattering media to channel light into the porous structure. In the porous bPBR, the microalgal cell attachment area in each region increased by 34.5 times. The maximum biomass yield of the porous biofilm PBR can reach 31.7 g m^−2^, promoting efficient microalgal growth. Tailored light exposure minimizes energy wastage by providing only the necessary light intensity and wavelengths required for photosynthesis, thereby improving overall energy efficiency in microalgal cultivation. However, as cell density increases, the light transmittance of 3D-printed bioreactors decreases and affects cell growth. Oh et al. (2023) designed 2 mm-thick plate-shaped engineering living materials placed on a solid plastic surface to prevent air transfer at the bottom. After 14 days of cultivation under blue and red light, the light transmittance attenuation increased from 62 and 43% to approximately 70% and 50%, respectively. However, the light transmittance attenuation worsened with the increasing cell density, indicating that it is a key factor limiting the industrial application of 3D-printed bioreactors.

### Increased biomass

Precision control of 3D printing bioreactor over the spatial distribution of microalgae allows for optimized growth conditions, leading to increased biomass yields. This method also enables the creation of intricate structures that enhance light exposure, nutrient absorption, and gas exchange, further promoting microalgal growth. 3D printing facilitates the design and customization of cultivation systems, contributing to resource efficiency and scalability in microalgal cultivation. In microalgal cultivation, light attenuation due to self-shading is a critical limiting factor for large-scale cultivation. Wangpraseurt et al. (2020) found that corals have an excellent light-enhancement system. By 3D printing biomimetic corals that mimic the coral–algae symbiotic system, microalgae can grow densely up to 10^^9^ cells mL^−1^. Huang et al. (2023) introduced silica photonic particles into photosensitive resin and used a 3D printer to create porous PBRs. This technique increased the spatial utilization and microalgae’s effective adsorption area by 3.13 times, resulting in a 32% improvement in microalgal cell adsorption capacity and a high growth rate of up to 3.572 g m^−2^ d^−1^ after a temperature increase. The following factors must be considered when implementing 3D printing for microalgal biomass enhancement: selecting suitable materials for the 3D printing matrix that promote microalgae growth, optimizing printing parameters to ensure cell viability, and addressing the scalability and cost-effectiveness of the technology for large-scale applications.

Assessment of biocompatibility between 3D printing materials and microalgae revealed that acrylate-methacrylate resin inhibits the growth of *C. vulgaris*, leading to its exemption as a material for photobioreactors (Pozzobon et al. 2023). A 3D micro-hydrocyclone separator was developed to efficiently harvest microalgae. In just 13 min of operation, the final microalgae biomass concentration increased by 11.1 times, overcoming the limitations of traditional microfluidic technologies (Syed et al. 2017). At low cell densities, 3D-printed bioreactors can effectively enhance cell biomass through improved mass transfer and tailored light exposure. However, at high cell densities, the limited carbon availability for the cells in deep layers becomes the primary factor restricting further biomass increase. Future studies should explore whether heterotrophic 3D-printed bioreactors can facilitate rapid organic carbon absorption.

### Enhanced photosynthesis

Custom-designed structures can optimize light exposure, improve light distribution within cultures, and provide a controlled environment for efficient photosynthetic activity. The successful implementation of 3D printing for enhancing photosynthesis requires careful consideration of various factors, such as selecting printing materials that are compatible with microalgal growth, optimizing structural designs for light penetration, and addressing the scalability and economic feasibility of the technology for large-scale photosynthetic enhancement. Oh et al. (2023) studied the growth and photosynthetic productivity of eukaryotic *Euglena gracilis* in 3D-shaped hydrogels and found that* E. gracilis* exhibited fast growth rates and high CO_2_ capture efficiency within the hydrogels. Zhao et al. (2019a) manufactured a high surface area-to-volume ratio fractal tree-like PBR for* C. vulgaris* cultivation using 3D printing. This PBR design achieved an F_v_/F_m_ at 0.767 and demonstrated high photosynthetic growth efficiency and CO_2_ capture efficiency. Traditional flat-panel PBRs have limitations in providing an adequate surface area for microalgae growth. 3D printing allows for the fabrication of complex structures with increased surface area, providing additional space for microalgal attachment and growth. In addition, 3D-printed structures can be engineered to optimize sunlight exposure, ensuring that the microalgae receive ideal lighting conditions for photosynthesis throughout their entire growth period (Zhao et al. 2019a).

## 3D printing for microalgal application

The integration of 3D printing with microalgae cultivation has paved the way for innovative applications, particularly in the development of microalgae-based bioreactors. This intersection offers a unique approach to design and customize bioreactor structures, optimizing conditions for microalgal growth and the production of valuable compounds (Table 3).

**Table 3:** Applications of 3D printing in various microalgal fields

	Application	Results	References
*Platymonas* sp.	Silk fibroin hydrogel	Supports growth for 4 weeks, photosynthesis for 90 days	(Zhao et al. 2019b)
*Chlamydomonas reinhardtii*	3D-printed hydrogel filter	Reduced copper concentration by 3%	(Thakare et al. 2021)
*Chlorella sorokiniana*	Preparation of 3D-printed porous photobioreactor	Increased adsorption area by 3.13 times; growth rate up to 3.572 g m^−2^ d^−1^	(Huang et al. 2023)
*Chlorella sorokiniana*	3D-printed porous biofilm photobioreactor (bPBR)	Increased attachment area by 34.5 times. Maximum biomass yield of 31.7 g m^−2^	(Zeng et al. 2023)
*Chlorella vulgaris, Arthrospira platensis*	3D-printed cereal snacks	Enhanced dough flow and printability	(Uribe-Wandurraga et al. 2020)
*Arthrospira platensis, Chlorella vulgaris*	3D-printed cookies	Improved extrudability, enhanced printability, and baking resistance	(Uribe-Wandurraga et al. 2021)
*Arthrospira platensis, Chlorella vulgaris*	Gluten-free cereal snacks	Higher antioxidant activity, vitamins, and nutrients	(Letras et al. 2022)
*Arthrospira platensis*	3D-printed cookies	Highest antioxidant activity and total phenolic content	(Vieira et al. 2020)
*Chlorella pyrenoidosa*	Novel photosynthetic live scaffold	Accelerates wound closure	(Wang et al. 2022)
*Chlamydomonas reinhardtii*	Alginate scaffold	16-fold increase in chlorophyll; oxygen production efficiency of 0.05 mg L^−1^ h^−1^	(Lode et al. 2015)
*Chlorella vulgaris*	Living carbon capture textile	Adsorbs CO_2_ from the environment	(Stefanova et al. 2021)
*Chlorella vulgaris, Spirulina platensis*	Sustainable fillers	Good printability and tensile strength	(Fiedler et al. 2023)
*Nannochloropsis salina*	PVA bio-based material filler	Increased stability	(Dang-Thuan et al. 2018)
*Pyrocystis lunula*	Hydrogel luminescent bio-material	Sensitive, high mechanical performance, used in biosensing	(Li et al. 2023)
*Chlamydomonas reinhardtii*	Alginate hydrogel bio-printing material	Used for creating artificial leaves and photosynthetic bio-clothing	(Balasubramanian et al. 2021)
*Chlorella vulgaris*	Encapsulated in carrageenan and clay composite hydrogel	Preparation of biocomposite ceramic substrate	(Crawford et al. 2022)

### Environmental remediation

Human activities are accelerating environmental degradation, necessitating measures to protect and restore polluted environments (Johnston et al. 2024). Photosynthetic autotrophic microalgae purify wastewater and absorb CO_2_ gas (Wang et al. 2023b, Wang et al. 2023a), playing a crucial role in mitigating environmental pressures. Utilizing 3D printing to create PBRs for microalgal growth enhances their environmental capabilities. For instance, Zhao et al. (2019b) used silk protein hydrogel materials to 3D print structures containing the marine microalgal strain *Platymonas* sp. These structures can support microalgal cell proliferation for at least 4 weeks, with photosynthetic activity lasting around 90 days. Maintaining long-term cell viability allows for improved oxygen supplementation and CO_2_ reduction, contributing to green indoor environments. Sung et al. (2022) used 3D printing to create buoyant structures that inhibit biofilm formation in PBRs, reducing microalgal biomass loss by 5.58%. Combined with CO_2_ for cultivation, this technique enhances microalgal productivity while reducing carbon emissions. Copper contamination poses severe risks in drinking water, and using microalgal species for copper removal is relatively cost-effective. Thakare et al. (2021) printed alginate hydrogel filters containing algal cells using an extrusion-based 3D printer. Compared with that when using traditional filters, the copper concentration in the test solution decreased by approximately 3% (from 0 ppm to 5.1 ppm) after filtering for 83 h, effectively removing copper. Through explorations on high-biocompatibility biological inks for 3D printing, microalgal capabilities in environmental management and air purification can be further enhanced.

### Food industry

Microalgae contain proteins, lipids, vitamins, minerals, and other nutrients, making them a novel food ingredient with broad nutritional value (Dhandwal et al. 2024). However, microalgae’s color and distinct odor limit their applications in the food industry. Uribe-Wandurraga et al. (2020) evaluated the potential of adding microalgae to cereal snacks using 3D printing and found that* C. vulgaris* and *Spirulina* enhanced the dough’s rheological properties and printability. Snacks with 3% and 4% *C. vulgaris* had the most accurate printing structures. In 3D-printed cookies, the incorporation of two microalgae biomasses into the dough increased the extrusion force, printability, and stability of the cookies’ 3D structure. As a novel food source, microalgae could address future food resource scarcity. Letras et al. (2022) used 3D printing to produce gluten-free cereal snacks combined with* C. vulgaris* and *Spirulina* biomasses and found that snacks with 5% *Spirulina* exhibited good nutritional and sensory characteristics, high antioxidant activity, and large amounts of protein and vitamins. These snacks could be introduced to the market as a novel sustainable snack. Vieira et al. (2020) incorporated *Dunaliella tertiolecta* into 3D-printed cookies as a biologically derived ink and found that extracts prepared with 0% ethanol and 2.0% biomass exhibited the highest antioxidant activity and total phenolic content. Incorporating this extract into printable cookie dough allows for the production of functional foods with antioxidant properties, further expanding the application of 3D printing in the food industry.

### Medical field

Microalgae contain pharmacologically active substances, such as phycobiliproteins, astaxanthin, carotenoids, and unsaturated fatty acids, making them valuable for pharmaceutical and healthcare product development. Owing to their photosynthetic autotrophic properties, they can also be used as bio dressings to provide oxygen to wounds and expedite healing. Inspired by the symbiosis of microalgae and salamanders, Wang et al. (2022) incorporated *C. vulgaris* into 3D-printed living photosynthetic scaffolds. Under light exposure, these scaffolds sustainably produced oxygen. When placed on the wounds of patients with diabetes, they significantly accelerated the closure of chronic wounds by alleviating local hypoxia, increasing angiogenesis, and promoting the synthesis of extracellular matrix. Lode et al. (2015) embedded *E. gracilis* in 3D-printed alginate scaffolds. The *E. gracilis* remained viable, with its chlorophyll content increasing 12-fold within 16 days under light exposure. The oxygen production efficiency reached 0.05 mg L^−1^ h^−1^ within 24 h, increasing over time. This coculture system paves the way for microalgal drug delivery.

### Novel material fabrication

Urban construction activities contribute to increased CO_2_ emissions. With accelerating material consumption, the development of new biodegradable and sustainable materials is urgently needed. Stefanova et al. (2021) 3D printed common* C. vulgaris* to create living photosynthetic carbon-capturing textiles for the construction industry. Microalgae feed on construction waste while absorbing CO_2_ from the indoor environment. Fiedler et al. (2023) integrated microalgae as a sustainable filler in long filament materials, mixing polyethylene-terephthalate-glycol with *Spirulina* and *Chlorella* to be extruded into 3D-printed filaments. The resulting filaments exhibited excellent printability and tensile strength, matching the performance of natural fillers such as wood and bamboo, making them suitable for low-cost sustainable material production. *Chlorella*-derived microalgae biomass was used as a filler to prepare polyvinyl alcohol (PVA) biocomposite materials. Although biomass addition reduced the mechanical performance of the composites compared with pure PVA, it enhanced the thermal stability. In addition, the introduction of poly (diallyldimethylammonium chloride) (PD) improved the mechanical performance. The biocomposite with 20% LEF and 12% PD had similar mechanical properties to pure PVA and an improved thermal performance (Li et al. 2023). Li et al. (2023) embedded diatom microalgae in hydrogel using 3D printing to develop an ultrasensitive luminescent biocomposite with robust mechanical properties. This novel material has a lifespan of 5 months in harsh environmental conditions and holds significant potential for applications in mechanical sensing, biosensing, and robotics. Balasubramanian et al. (2021) used an eco-friendly chemical approach to encapsulate microalgae within an alginate hydrogel matrix, which was then 3D-printed into biocompatible materials with sufficient mechanical strength. These renewable living materials can survive for 3 days without any nutrients and extend their lifespan when transferred to fresh nutrient sources. They have potential applications in artificial leaves and photosynthetic biofabric. Crawford et al. (2022) encapsulated diatoms in a hydrogel composed of carrageenan and clay binder to prepare ceramic substrates for construction, laying the foundation for the development of ceramic-based composites.

## Challenges and future perspectives

The application of 3D printing in microalgal bioreactors shows promising prospects. As this technology continues to evolve and be optimized, its applications in microalgae cultivation, environmental stress alleviation, and product manufacturing will become extensive. The use of 3D printing in microalgal bioreactors will also drive technological innovation and industrial upgrading in related fields, providing robust support for sustainable development and a green economy. However, the current application of 3D printing in microalgal bioreactors still faces several challenges, such as the selection of printing materials, regulation of cultivation conditions, and reduction of production costs. To overcome these issues, future research should focus on the following areas: (a) develop high-performance 3D printing materials to enhance the practicality and durability of bioreactors, (b) optimize cultivation conditions in printed bioreactors to increase the growth rate and microalgal biomass, (c) reduce the cost of 3D printing bioreactors to enable large-scale production and application, (d) explore integration with other bioreactor technologies to achieve targeted cultivation and efficient production of microalgae, and (e) conduct research on industrial applications to promote the sustainable development of the microalgal industry.

## Conclusion

3D printing technology for microalgal bioreactors showcases a revolutionary approach to enhance microalgal production and combat environmental challenges. This technology offers precision in design, optimizing growth conditions and contributing to carbon capture. The versatility of 3D printing allows tailored solutions for diverse microalgae species, promoting their adaptability to adverse conditions. By aligning with circular economy principles, this innovation fosters sustainability and resource efficiency. As an evolving field, 3D-printed bioreactors hold immense promise for greening various microalgal industries, from food to bioremediation.

## Data Availability

Not applicable.

## References

[CR1] Alcala-Orozco CR, Mutreja I, Cui X, Hooper GJ, Lim KS, Woodfield TBF. Hybrid biofabrication of 3D osteoconductive constructs comprising Mg-based nanocomposites and cell-laden bioinks for bone repair. Bone. 2022;154:116198. 34534709 10.1016/j.bone.2021.116198

[CR2] Amorim ML, Soares J, dos Reis Coimbra JS, Leite MdO, Teixeira Albino LF, Martins MA. Microalgae proteins: production, separation, isolation, quantification, and application in food and feed. Crit Rev Food Sci. 2021;61:1976–2002. 10.1080/10408398.2020.1768046 32462889

[CR3] Balasubramanian S, Yu K, Meyer AS, Karana E, Aubin-Tam M-E. Bioprinting of Regenerative Photosynthetic Living Materials. Adv Funct Mater. 2021;31:2011162.

[CR4] Branyikova I, Lucakova SJOA. Technical and physiological aspects of microalgae cultivation and productivity—spirulina as a promising and feasible choice. Org Agr. 2021;11:269–76.

[CR5] Caporgno MP, Mathys A. Trends in Microalgae Incorporation Into Innovative Food Products With Potential Health Benefits. Front Nutr. 2018;5:58. 30109233 10.3389/fnut.2018.00058 PMC6080594

[CR6] Carlsson AS, Van Bilein JB, Möller R, Clayton D, Bowles D. Output from EPOBIO Project: Micro- and Macroalgae Utility for Industrial Application. York, U.K.: CPL Press; 2007. p. 1–86.

[CR7] Chang HK, Yang DH, Ha MY, Kim HJ, Kim CH, Kim SH, Choi JW, Chun HJ. 3D printing of cell-laden visible light curable glycol chitosan bioink for bone tissue engineering. Carbohyd Polym. 2022;287:119328. 10.1016/j.carbpol.2022.119328 35422276

[CR8] Choi DJ, Choi K, Park SJ, Kim Y-J, Chung S, Kim C-H. Suture Fiber Reinforcement of a 3D Printed Gelatin Scaffold for Its Potential Application in Soft Tissue Engineering. Int J Mol Sci. 2021;22:11600. 34769034 10.3390/ijms222111600 PMC8584198

[CR9] Crawford A, In-na P, Caldwell G, Armstrong R, Bridgens B. Clay 3D printing as a bio-design research tool: development of photosynthetic living building components. Archit Sci Rev. 2022;65:185–95.

[CR10] Dang-Thuan T, Lee HR, Jung S, Park MS, Yang J-W. Lipid-extracted algal biomass based biocomposites fabrication with poly (vinyl alcohol). Algal Res. 2018;31:525–33.

[CR11] Datta P, Sarkar R, Vyas V, Bhutoria S, Barui A, Chowdhury A, Datta P. Alginate-honey bioinks with improved cell responses for applications as bioprinted tissue engineered constructs. J Mater Res. 2018;2018(33):2029–39.

[CR12] Dhandwal A, Bashir O, Malik T, et al. Sustainable microalgal biomass as a potential functional food and its applications in food industry: a comprehensive review. Environ Sci Pollut Res. 2024. 10.1007/s11356-024-33431-6. 10.1007/s11356-024-33431-6 38710849

[CR13] Dwivedi R, Mehrotra D. 3D bioprinting and craniofacial regeneration. J Oral Biol Craniofac Res. 2020;10:650–9. 32983859 10.1016/j.jobcr.2020.08.011 PMC7493084

[CR14] Fiedler M, Schoemig O, Fischer F, Droeder K. Technological Evaluation of Algae-Based Fillers for Polymer 3D Printing. Sustainability. 2023;15:4039.

[CR15] Gudapati H, Dey M, Ozbolat I. A comprehensive review on droplet-based bioprinting: Past, present and future. Biomaterials. 2016;102:20–42. 27318933 10.1016/j.biomaterials.2016.06.012

[CR16] Hao L, Zhao S, Hao S, He Y, Feng M, Zhou K, He Y, Yang J, Mao H, Gu Z. Functionalized gelatin-alginate based bioink with enhanced manufacturability and biomimicry for accelerating wound healing. Int J Biol Macromol. 2023;240:124364. 37044319 10.1016/j.ijbiomac.2023.124364

[CR17] He Y, Derakhshanfar S, Zhong W, Li B, Lu F, Xing M, Li X. Characterization and application of carboxymethyl chitosan-based bioink in cartilage tissue engineering. J Nanomater. 2020;2020:2057097.

[CR18] Hepburn C, Adlen E, Beddington J, Carter EA, Fuss S, Mac Dowell N, Minx JC, Smith P, Williams CK. The technological and economic prospects for CO2 utilization and removal. Nature. 2019;575:87–97. 31695213 10.1038/s41586-019-1681-6

[CR19] Huang Y, Zhanga B, Chena K, Xiaa A, Zhua X, Zhua X, Liao Q. Temperature-controlled microalgae biofilm adsorption/desorption in a thermo-responsive light-guided 3D porous photo-bioreactor for CO2 fixation. Environ Res. 2023;216:114645. 36323351 10.1016/j.envres.2022.114645

[CR20] Im S, Choe G, Seok JM, Yeo SJ, Lee JH, Kim WD, Lee JY, Park SA. An osteogenic bioink composed of alginate, cellulose nanofibrils, and polydopamine nanoparticles for 3D bioprinting and bone tissue engineering. Int J Biol Macromol. 2022;205:520–9. 35217077 10.1016/j.ijbiomac.2022.02.012

[CR21] Johnston FH, Williamson G, Borchers-Arriagada N, Henderson SB, Bowman DMJS. Climate Change, Landscape Fires, and Human Health: A Global Perspective. Annu Rev Publ Health. 2024;45:295–314. 10.1146/annurev-publhealth-060222-034131 38166500

[CR22] Khoeini R, et al. Natural and Synthetic Bioinks for 3D Bioprinting. Adv Nanobiomed Res. 2021;1:2000097.

[CR23] Kim YB, Lee H, Yang G-H, Choi CH, Lee D, Hwang H, Jung W-K, Yoon H, Kim GH. Mechanically reinforced cell-laden scaffolds formed using alginate-based bioink printed onto the surface of a PCL/alginate mesh structure for regeneration of hard tissue. J Colloid Interf Sci. 2016;461:359–68. 10.1016/j.jcis.2015.09.044 26409783

[CR24] Kostenko A, Connon CJ, Swioklo S. Storable Cell-Laden Alginate Based Bioinks for 3D Biofabrication. Bioengineering. 2023;10:23. 10.3390/bioengineering10010023 PMC9854877 36671596

[CR25] Kulkarni VR, Saha T, Giri BR, Lu AQ, Das SC, Maniruzzaman M. Recent Advancements in Pharmaceutical 3D Printing Industry. J Drug Deliv Sci Tec. 2024;100:106072.

[CR26] Lee JS, Sung YJ, Sim SJ. Kinetic analysis of microalgae cultivation utilizing 3D-printed real-time monitoring system reveals potential of biological CO_2_ conversion. Bioresource Technol. 2022;364:128014. 10.1016/j.biortech.2022.128014 36155817

[CR27] Letras P, Oliveira S, Varela J, Nunes MC, Raymundo A. 3D printed gluten-free cereal snack with incorporation of Spirulina (Arthrospira platensis) and/or Chlorella vulgaris. Algal Res. 2022;68:102863.

[CR28] Li C, Schramma N, Wang Z, Qari NF, Jalaal M, Latz MI, Cai S. Ultrasensitive and robust mechanoluminescent living composites. Sci Adv. 2023;9:eadi8643. 37862415 10.1126/sciadv.adi8643 PMC10588950

[CR29] Ligon SC, Liska R, Stampfl J, Gurr M, Muelhaupt R. Polymers for 3D Printing and Customized Additive Manufacturing. Chem Rev. 2017;117:10212–90. 28756658 10.1021/acs.chemrev.7b00074 PMC5553103

[CR30] Liu H, et al. Space-Efficient 3D Microalgae Farming with Optimized Resource Utilization for Regenerative Food. Adv Mater. 2024; e2401172-e2401172. 10.1002/adma.202401172 38483347

[CR31] Lode A, Krujatz F, Brueggemeier S, Quade M, Schuetz K, Knaack S, Weber J, Bley T, Gelinsky M. Green bioprinting: Fabrication of photosynthetic algae-laden hydrogel scaffolds for biotechnological and medical applications. Eng Life Sci. 2015;15:177–83.

[CR32] Murphy SV, Atala A. 3D bioprinting of tissues and organs. Nat Biotechnol. 2014;32:773–85. 25093879 10.1038/nbt.2958

[CR33] Nagarajan D, Varjani S, Lee DJ, Chang JS. Sustainable aquaculture and animal feed from microalgae-Nutritive value and techno-functional components. Renew Sust Energ Rev. 2021;150:111549.

[CR34] Ng WL, Yeong WY, Naing MW. Polyvinylpyrrolidone-Based Bio-Ink Improves Cell Viability and Homogeneity during Drop-On-Demand Printing. Materials. 2017;10:190. 28772551 10.3390/ma10020190 PMC5459162

[CR35] Oh J-J, Ammu S, Vriend VD, Kieffer R, Kleiner FH, Balasubramanian S, Karana E, Masania K, Aubin-Tam M-E. Growth, Distribution, and Photosynthesis of Chlamydomonas Reinhardtii in 3d Hydrogels. Adv Mater. 2023; e2305505-e2305505. 10.1002/adma.202305505 37851509

[CR36] Pahlevanzadeh F, Mokhtari H, Bakhsheshi-Rad HR, Emadi R, Kharaziha M, Valiani A, Poursamar SA, Ismail AF, RamaKrishna S, Berto F. Recent Trends in Three-Dimensional Bioinks Based on Alginate for Biomedical Applications. Materials. 2020;13:3980. 32911867 10.3390/ma13183980 PMC7557490

[CR37] Pant S, Thomas S, Loganathan S, Valapa RB. 3D bioprinted poly(lactic acid)/mesoporous bioactive glass based biomimetic scaffold with rapid apatite crystallization and in-vitro Cytocompatability for bone tissue engineering. Int J Biol Macromol. 2022;217:979–97. 35908677 10.1016/j.ijbiomac.2022.07.202

[CR38] Parra-Cabrera C, Achille C, Kuhn S, Ameloot R. 3D printing in chemical engineering and catalytic technology: structured catalysts, mixers and reactors. Chem Soc Rev. 2018;47:209–30. 29131228 10.1039/c7cs00631d

[CR39] Peak CW, Stein J, Gold KA, Gaharwar AK. Nanoengineered colloidal inks for 3D bioprinting. Langmuir. 2018;34:917–25. 28981287 10.1021/acs.langmuir.7b02540 PMC12861117

[CR40] Piluso S, Skvortsov GA, Altunbek M, Afghah F, Khani N, Koc B, Patterson J. 3D bioprinting of molecularly engineered PEG-based hydrogels utilizing gelatin fragments. Biofabrication. 2021;13:045008. 10.1088/1758-5090/ac0ff0 34192670

[CR41] Pozzobon V, Otaola F, Arnoudts C, Lagirarde J. Impact of 3D printing materials on mircoalga Chlorella vulgaris. Bioresource Technol. 2023;389:129807. 10.1016/j.biortech.2023.129807 37778670

[CR42] Raees S, et al. Classification, processing, and applications of bioink and 3D bioprinting: A detailed review. Int J Biol Macromol. 2023;232:123476. 36731696 10.1016/j.ijbiomac.2023.123476

[CR43] Sadeghianmaryan A, Naghieh S, Yazdanpanah Z, Sardroud HA, Sharma NK, Wilson LD, Chen X. Fabrication of chitosan/alginate/hydroxyapatite hybrid scaffolds using 3D printing and impregnating techniques for potential cartilage regeneration. Int J Biol Macromol. 2022;204:62–75. 35124017 10.1016/j.ijbiomac.2022.01.201

[CR44] Sathish PB, Gayathri S, Priyanka J, Muthusamy S, Narmadha R, Shankar KG, Selvakumar R. Tricomposite gelatin-carboxymethylcellulose-alginate bioink for direct and indirect 3D printing of human knee meniscal scaffold. Int J Biol Macromol. 2022;195:179–89. 34863969 10.1016/j.ijbiomac.2021.11.184

[CR45] Shahrubudin N, Lee TC, Ramlan R. An Overview on 3D Printing Technology: Technological, Materials, and Applications, 2nd International Conference on Sustainable Materials Processing and Manufacturing (SMPM), South Africa, 2019; pp. 1286–1296.

[CR46] Shamma RN, Sayed RH, Madry H, El Sayed NS, Cucchiarini M. Triblock Copolymer Bioinks in Hydrogel Three-Dimensional Printing for Regenerative Medicine: A Focus on Pluronic F127. Tissue Eng Part B-Re. 2022;28:451–63. 10.1089/ten.TEB.2021.0026 33820451

[CR47] Shopperly LK, Spinnen J, Krueger J-P, Endres M, Sittinger M, Lam T, Kloke L, Dehne T. Blends of gelatin and hyaluronic acid stratified by stereolithographic bioprinting approximate cartilaginous matrix gradients. J Biomed Mater Res. 2022;110:2310–22. 10.1002/jbm.b.35079 35532378

[CR48] Soman P, Kelber JA, Lee JW, Wright TN, Vecchio KS, Klemke RL, Chen S. Cancer cell migration within 3D layer-by-layer microfabricated photocrosslinked PEG scaffolds with tunable stiffness. Biomaterials. 2012;33:7064–70. 22809641 10.1016/j.biomaterials.2012.06.012 PMC3420339

[CR49] Stefanova A, In-na P, Caldwell GS, Bridgens B, Armstrong R. Photosynthetic textile biocomposites: Using laboratory testing and digital fabrication to develop flexible living building materials. Sci Eng Compos Mater. 2021;28:223–36.

[CR50] Sun H, Wang Y, He Y, Liu B, Mou H, Chen F, Yang S. Microalgae-Derived Pigments for the Food Industry. Mar Drugs. 2023;21:82. 36827122 10.3390/md21020082 PMC9967018

[CR51] Sung YJ, Yoon HK, Lee JS, Joun J, Yu BS, Sirohi R, Sim SJ. Novel 3D-printed buoyant structures for improvement in flue gas CO2-derived microalgal biomass production by enhancing anti-biofouling on vertical polymeric photobioreactor. J Clean Prod. 2022;366:133030.

[CR52] Syed MS, Rafeie M, Henderson R, Vandamme D, Asadnia M, Warkiani ME. A 3D-printed mini-hydrocyclone for high throughput particle separation: application to primary harvesting of microalgae. Lab Chip. 2017;17:2459–69. 28695927 10.1039/c7lc00294g

[CR53] Thakare K, Jerpseth L, Pei Z, Tomlin B, Qin H. Three-Dimensional Printing of Hydrogel Filters Containing Algae Cells for Copper Removal From Contaminated Water. J Manuf Sci E-T Asme. 2021;143:104502.

[CR54] Tian X, Jin J, Yuan S, Chua CK, Tor SB, Zhou K. Emerging 3D-Printed Electrochemical Energy Storage Devices: A Critical Review. Adv Energy Mater. 2017;7:1700127.

[CR55] Trager A, Naeimipour S, Jury M, Selegard R, Aili D. Nanocellulose Reinforced Hyaluronan-Based Bioinks. Biomacromol. 2023;24:3086–93. 10.1021/acs.biomac.3c00168 PMC10336840 37341704

[CR56] Tripathi S, Mandal SS, Bauri S, Maiti P. 3D bioprinting and its innovative approach for biomedical applications. Medcomm. 2023;4:e194. 36582305 10.1002/mco2.194 PMC9790048

[CR57] Uehlin AF. Optimization of a biomimetic poly-(lactic acid) ligament scaffold. 2012; The University of Alabama at Birmingham.

[CR58] Uribe-Wandurraga ZN, Zhang L, Noort MWJ, Schutyser MAI, Garcia-Segovia P, Martinez-Monzo J. Printability and Physicochemical Properties of Microalgae-Enriched 3D-Printed Snacks. Food Bioprocess Tech. 2020;13:2029–42.

[CR59] Uribe-Wandurraga ZN, Igual M, Reino-Moyon J, Garcia-Segovia P, Martinez-Monzo J. Effect of Microalgae (Arthrospira platensis and Chlorella vulgaris) Addition on 3D Printed Cookies. Food Biophys. 2021;16:27–39.

[CR60] Vieira MV, Oliveira SM, Amado IR, Fasolin LH, Vicente AA, Pastrana LM, Fucinos P. 3D printed functional cookies fortified with Arthrospira platensis: Evaluation of its antioxidant potential and physical-chemical characterization. Food Hydrocolloid. 2020;107:105893.

[CR61] Wang X, Yang C, Yu Y, Zhao Y. In Situ 3D Bioprinting Living Photosynthetic Scaffolds for Autotrophic Wound Healing. Research. 2022;2022:9794745. 35387266 10.34133/2022/9794745 PMC8961369

[CR62] Wang Y, Yang S, Liu J, Wang J, Xiao M, Liang Q, Ren X, Wang Y, Mou H, Sun H. Realization process of microalgal biorefinery: The optional approach toward carbon net-zero emission. Sci Total Environ. 2023b;901:165546–165546. 37454852 10.1016/j.scitotenv.2023.165546

[CR63] Wang J, Wang Y, Gu Z, Mou H, Sun H. Stimulating carbon and nitrogen metabolism of Chlorella pyrenoidosa to treat aquaculture wastewater and produce high-quality protein in plate photobioreactors. Sci Total Environ. 2023b;878:163061. 36963682 10.1016/j.scitotenv.2023.163061

[CR64] Wang J, Wang Y, Xiao M, Liang Q, Yang S, Liu J, Zhang Y, Mou H, Sun H. Upcycling food waste into biorefinery production by microalgae. Chem Eng J. 2024a;484:149532.

[CR65] Wang Y, Wang J, Yang S, Liang Q, Gu Z, Wang Y, Mou H, Sun H. Selecting a preculture strategy for improving biomass and astaxanthin productivity of Chromochloris zofingiensis. Appl Microbiol Biot. 2024b;108:18–18. 10.1007/s00253-023-12873-x PMC10781847 38204137

[CR66] Wang H, Bi S, Shi B, Ma J, Lv X, Qiu J, Wei Y. Recent Advances in Engineering Bioinks for 3D Bioprinting. Adv Eng Mater. 2023a; 25.

[CR67] Wangpraseurt D, et al. Bionic 3D printed corals. Nat Commun. 2020;11:1748. 32273516 10.1038/s41467-020-15486-4 PMC7145811

[CR68] Wangpraseurt D, You S, Sun Y, Chen S. Biomimetic 3D living materials powered by microorganisms. Trends Biotechnol. 2022;40:843–57. 35115172 10.1016/j.tibtech.2022.01.003

[CR69] Wu X, Chen K, Chai Q, Liu S, Feng C, Xu L, Zhang D. Freestanding vascular scaffolds engineered by direct 3D printing with Gt-Alg-MMT bioinks. Biomater Adv. 2022;133:112658–112658. 35067435 10.1016/j.msec.2022.112658

[CR70] Yang Y, Wang Z, Xu Y, Xia J, Xu Z, Zhu S, Jin M. Preparation of Chitosan/Recombinant Human Collagen-Based Photo-Responsive Bioinks for 3D Bioprinting. Gels. 2022;8:314. 35621612 10.3390/gels8050314 PMC9141723

[CR71] Yang S, Wang Y, Wang J, Cheng K, Liu J, He Y, Zhang Y, Mou H, Sun H. Microalgal protein for sustainable and nutritious foods: A joint analysis of environmental impacts, health benefits and consumer’s acceptance. Trends Food Sci Tech. 2024;143:104278.

[CR72] Yang S, Fan Y, Cao Y, Wang Y, Mou H, Sun H. Technological readiness of commercial microalgae species for foods. Crit Rev Food Sci. 2023; 1–25. 10.1080/10408398.2023.2194423 36999969

[CR73] Yarnold J, Karan H, Oey M, Hankamer B. Microalgal Aquafeeds As Part of a Circular Bioeconomy. Trends Plant Sci. 2019;24:959–70. 31285128 10.1016/j.tplants.2019.06.005

[CR74] Yu Z, Zhao W, Sun H, Mou H, Liu J, Yu H, Dai L, Kong Q, Yang S. Phycocyanin from microalgae: A comprehensive review covering microalgal culture, phycocyanin sources and stability. Food Res Int. 2024;186:114362. 38729724 10.1016/j.foodres.2024.114362

[CR75] Zeng W, Chen K, Huang Y, Xia A, Zhu X, Zhu X, Liao Q. Three-dimensional porous biofilm photobioreactor with light-conducting frameworks for high-efficiency microalgal growth. Algal Res. 2023;69:102942.

[CR76] Zhao L, Zeng G, Gu Y, Tang Z, Wang G, Tang T, Shan Y, Sun Y. Nature inspired fractal tree-like photobioreactor via 3D printing for CO2 capture by microaglae. Chem Eng Sci. 2019a;193:6–14.

[CR77] Zhao S, Guo C, Kumarasena A, Omenetto FG, Kaplan DL. 3D Printing of Functional Microalgal Silk Structures for Environmental Applications. ACS Biomater Sci Eng. 2019b;5:4808–16. 33448823 10.1021/acsbiomaterials.9b00554

[CR78] Zheng Z, Wu J, Liu M, Wang H, Li C, Rodriguez MJ, Li G, Wang X, Kaplan DL. 3D Bioprinting of Self-Standing Silk-Based Bioink. Adv Healthc Mater. 2018;7:1701026. 10.1002/adhm.201701026 29292585

[CR79] Zhu W, Ma X, Gou M, Mei D, Zhang K, Chen S. 3D printing of functional biomaterials for tissue engineering. Curr Opin Biotech. 2016;40:103–12. 27043763 10.1016/j.copbio.2016.03.014

[CR80] Zou S, Fan S, Oliveira AL, Yao X, Zhang Y, Shao H. 3D Printed Gelatin Scaffold with Improved Shape Fidelity and Cytocompatibility by Using Antheraea pernyi Silk Fibroin Nanofibers. Adv Fiber Mater. 2022;4:758–73.

